# Environmental determinants of mental health in clinical practice

**DOI:** 10.1192/j.eurpsy.2024.84

**Published:** 2024-08-27

**Authors:** K. Catthoor

**Affiliations:** ^1^CAPRI, University of Antwerp, Antwerp; ^2^Flemish Association of Psychiatry, Kortenberg; ^3^Psychiatry, Ziekenhuis Netwerk Antwerpen, Antwerp, Belgium

## Abstract

**Abstract:**

According to the latest Intergovernmental Panel on Climate Change (IPCC) Report (2022), climate changes (e.g. rising sea levels and temperatures and) are noticeable and intensifying on the entire planet. Extreme weather events or ecological disasters are occurring with increased frequency and intensity. Anthropogenic climate change has been called “the defining issue of our time” (United Nations, 2022) and “the greatest threat to global health in the 21st century” (World Health Organization, 2015). Health impacts from climate change may include increased morbidity and mortality from worsening cardiopulmonary health, and greater risk of infectious diseases and mental illness. During this lecture, we will discuss environmental aspects that clearly have a negative impact on the mental well-being of the general population and, more specifically, the psychiatric population. The focus will primarily delve deeper into climate anxiety.

**Disclosure of Interest:**

None Declared

